# Global Genomic Epidemiology of Salmonella enterica Serovar Typhimurium DT104

**DOI:** 10.1128/AEM.03821-15

**Published:** 2016-04-04

**Authors:** Pimlapas Leekitcharoenphon, Rene S. Hendriksen, Simon Le Hello, François-Xavier Weill, Dorte Lau Baggesen, Se-Ran Jun, David W. Ussery, Ole Lund, Derrick W. Crook, Daniel J. Wilson, Frank M. Aarestrup

**Affiliations:** a Research Group for Genomic Epidemiology, National Food Institute, Technical University of Denmark, Kgs. Lyngby, Denmark; b Center for Biological Sequence Analysis, Department of System Biology, Technical University of Denmark, Kgs. Lyngby, Denmark; c Institut Pasteur, Unité des Bactéries Pathogènes Entériques, Centre National de Référence des Salmonella, Paris, France; d Technical University of Denmark, National Food Institute, Søborg, Denmark; e Comparative Genomics Group, Biosciences Division, Oak Ridge National Laboratory, Oak Ridge, Tennessee, USA; f Nuffield Department of Medicine, University of Oxford, John Radcliffe Hospital, Oxford, United Kingdom; g Wellcome Trust Centre for Human Genetics, University of Oxford, Oxford, United Kingdom; Tartu University

## Abstract

It has been 30 years since the initial emergence and subsequent rapid global spread of multidrug-resistant Salmonella enterica serovar Typhimurium DT104 (MDR DT104). Nonetheless, its origin and transmission route have never been revealed. We used whole-genome sequencing (WGS) and temporally structured sequence analysis within a Bayesian framework to reconstruct temporal and spatial phylogenetic trees and estimate the rates of mutation and divergence times of 315 *S*. Typhimurium DT104 isolates sampled from 1969 to 2012 from 21 countries on six continents. DT104 was estimated to have emerged initially as antimicrobial susceptible in ∼1948 (95% credible interval [CI], 1934 to 1962) and later became MDR DT104 in ∼1972 (95% CI, 1972 to 1988) through horizontal transfer of the 13-kb Salmonella genomic island 1 (SGI1) MDR region into susceptible strains already containing SGI1. This was followed by multiple transmission events, initially from central Europe and later between several European countries. An independent transmission to the United States and another to Japan occurred, and from there MDR DT104 was probably transmitted to Taiwan and Canada. An independent acquisition of resistance genes took place in Thailand in ∼1975 (95% CI, 1975 to 1990). In Denmark, WGS analysis provided evidence for transmission of the organism between herds of animals. Interestingly, the demographic history of Danish MDR DT104 provided evidence for the success of the program to eradicate Salmonella from pig herds in Denmark from 1996 to 2000. The results from this study refute several hypotheses on the evolution of DT104 and suggest that WGS may be useful in monitoring emerging clones and devising strategies for prevention of Salmonella infections.

## INTRODUCTION

Salmonella is one of the most common food-borne pathogens worldwide (1). In the United States of America alone, Salmonella is estimated to cause 1.4 million cases of salmonellosis, resulting in 17,000 hospitalizations and almost 600 deaths each year (2, 3). Globally, Salmonella enterica serovar Typhimurium is the most commonly isolated serovar (1). *S*. Typhimurium consists of a number of subtypes that classically have been divided by phage typing. During the last 3 decades, *S*. Typhimurium phage type DT104 (DT104) emerged as the most important phage type and one of the best studied because of its rapid global dissemination (1, 4). One of the characteristics of DT104 is its typical resistance to ampicillin, chloramphenicol, streptomycin, sulfonamide, and tetracycline (ACSSuT) (5) along with its capacity to acquire additional resistance to other clinically important antimicrobials (4).

Susceptible DT104 was first reported in the 1960s in humans and subsequently as multidrug-resistant DT104 (MDR DT104) in the early 1980s in humans and birds from the United Kingdom (6–9). Another instance of human MDR DT104 was reported in Hong Kong in the late 1970s (10). Isolates from agricultural animals were first reported in the United Kingdom in 1988 (8) and in the United States in 1990 (11). MDR DT104 rapidly emerged globally in the 1990s and became the most prevalent phage type isolated from humans and animals in many countries (4, 6, 12). Previous epidemics with MDR phage types of *S*. Typhimurium, such as DT29, DT204, DT193, and DT204c, were mostly restricted to cattle, whereas MDR DT104 spread among all domestic animals, including cattle, poultry, pigs, and sheep (6). A decline in MDR DT104 has been reported in the last decade (13, 14).

A recent study used whole-genome sequencing (WGS) to study DT104 mainly from cattle and humans in Scotland (15). This study was hampered by the lack of inclusion of isolates from other animal species and from food products consumed in Scotland but imported from other countries (15, 16).

The origin and transmission routes of the phage type DT104 are still ambiguous. Based on the presence of the rare resistance genes *floR* and *tet*(G), it has been suggested that the MDR phage type originated in Southeast Asia (6). Transmission has been suggested to be through trade of live animals, but it has not been established whether the epidemiologies in the different animal species are part of a common global spread or whether there are host-specific variants.

In order to examine the population structure of DT104, we sequenced a carefully selected representative intercontinental DT104 collection from different human and animal sources in 21 countries, covering the period from 1969 to 2012. We identified single nucleotide polymorphisms (SNPs) and performed phylogenomic dating based on temporally structured sequence analysis within a Bayesian framework in order to characterize the population structure, phylogeny, and evolution over time of DT104. We also revealed historical as well as recent dissemination events, including local transmission between and within farms in Denmark.

## MATERIALS AND METHODS

### Bacterial isolates.

The 315 *S*. Typhimurium DT104 isolates included in this study were collected intercontinentally from 21 countries: Argentina (*n* = 5), Austria (*n* = 30), Canada (*n* = 6), Czech Republic (*n* = 9), Denmark (*n* = 79), France (*n* = 9), Germany (*n* = 27), Ireland (*n* = 10), Israel (*n* = 17), Japan (*n* = 10), Luxembourg (*n* = 13), Morocco (*n* = 2), The Netherlands (*n* = 22), New Zealand (*n* = 7), Poland (*n* = 13), Scotland (*n* = 14), Spain (*n* = 1), Switzerland (*n* = 8), Taiwan (*n* = 13), Thailand (*n* = 8), and the United States (*n* = 12). All isolates from Japan and Scotland were retrieved as paired-end reads from the recent study (15) via the European Nucleotide Archive (ENA). The other isolates were supplied from the laboratory strain collections in the respective countries. The collection dates of the isolates ranged from 1969 to 2012; the oldest isolates were a horse isolate from France in 1969, human isolates from Morocco in 1975 and 1981, and a human isolate from Spain in 1976. Isolates were sampled from various sources: cattle (*n* = 35), poultry (*n* = 51), swine (*n* = 109), a hare (*n* = 1), a horse (*n* = 1), and humans (*n* = 118). The full details for the isolates used in this study are shown in Data Set S1 in the supplemental material.

### Whole-genome sequencing, *de novo* assembly, and resistance genes.

Isolates were sequenced using either the Illumina HiSeq or the MiSeq platform. Raw sequence data have been submitted to the European Nucleotide Archive (ENA). The raw reads were *de novo* assembled using the pipeline available from the Center for Genomic Epidemiology (CGE) (www.genomicepidemiology.org), which is based on Velvet algorithms, for the *de novo* short read assembly (17). A complete list of genomic sequence data is available in Data Set S1. The assembled genomes were analyzed using similar pipelines available on the CGE website. The webserver ResFinder (18) was used to detect acquired antimicrobial resistance genes with a selected threshold equal to 80% identity.

### Core genes.

Protein sequences were clustered based on sequence similarities by employing the Markov clustering algorithm (MCL) (19), a network-based unsupervised clustering algorithm. To generate an undirected network of protein sequences for input into the MCL, we first did all-against-all BLAST using an E value of 0.0001 and BLASTp (20) and kept only pairs of proteins whose reciprocal alignments removed gaps that are at least 50% as long as their query sequences and have at least 50% sequence identity. The network was generated by connecting proteins in conserved pairs with weight defined as the maximum sequence identity between reciprocal alignments where the sequence identity of alignments was adjusted along query sequences. The core genome was built from the intersection of gene clusters shared by every genome under analysis (21).

### SNP identification.

Single nucleotide polymorphisms (SNPs) were determined using CSI Phylogeny 1.1, available from the Center for Genomic Epidemiology (www.genomicepidemiology.org) (22, 23). Fundamentally, the pipeline consists of various publicly available programs. The paired-end reads were aligned against the reference genome, *S*. Typhimurium DT104 (GenBank accession number HF937208, genome length 4,933,631 bp) (15), using the Burrows-Wheeler aligner (BWA) (24). SAMtools (25) mpileup commands were used to identify and filter SNPs. The qualified SNPs were selected once they met the following criteria: (i) a minimum coverage (the number of reads mapped to reference positions) of 5, (ii) a minimum distance of 15 bp between each SNP, (iii) a minimum quality score for each SNP at 20, and (iv) the exclusion of all indels. The final qualified SNPs for each genome were concatenated to an alignment by an in-house Python script. The SNP alignments were subjected to maximum-likelihood tree construction using PhyML (26).

### Recombination detection.

SNP alignments had been detected for significant recombination sites prior to reconstruction of the phylogenetic trees. We used a novel hidden Markov model tool called RecHMM (27) to detect the clusters of sequence diversity that mark the recombination events within branches. RecHMM is computationally more practical than ClonalFrame (28) and yields comparable results.

### Temporal Bayesian phylogeny, discrete phylogeographic analysis, and Bayesian skyline plot.

SNP alignments were analyzed with BEAST (Bayesian evolutionary analysis sampling trees), version 1.7 (29, 30) for temporal phylogenetic reconstruction and estimations of mutation rates and divergence times. Several combinations of population size change and molecular clock models were evaluated to find the best-fit models. Among the models tested, the combination of a skyline model (31) of population size change and a relaxed uncorrelated log-normal clock gave the highest Bayes factors. The model that was selected allows the evolutionary rates to change (32) among the branches of the tree and has a general time-reversible (GTR) substitution model with γ correction for among-site rate variations.

All BEAST Monte Carlo Markov chains (MCMC) were run for at least 150 million and up to 300 million steps, with subsampling every 10,000 steps. The trees produced by BEAST were summarized by a single maximum clade credibility (MCC) tree using TreeAnnotator (30) with 10% of the MCMC steps discarded as burn-in. Statistical uncertainty was represented by a 95% credible interval (CI) calculated as the 95% highest posterior density (HPD) interval. A final tree was visualized and edited in FigTree (http://tree.bio.ed.ac.uk/software/figtree/). The geographic locations and direction of the transmissions were estimated by discrete phylogeographic analysis using a standard continuous-time Markov chain (CTMC) (33) implemented in BEAST. A location-annotated MCC tree was converted to the KML format using phylogeo.jar, which is relatively equivalent to SPREAD (http://www.phylogeography.org/SPREAD.html). The KML file was then visualized in Google Earth (http://earth.google.com/).

The demographic history was reconstructed using the Bayesian skyline plot implemented in Tracer (30) by processing the inferred genealogy and effective population size estimated by BEAST at different points along the genealogy time scale. The population size was inferred by the product of the interval size (γ_*i*_) and *i*(*i* − 1)/2, where *i* is the number of genealogical lineages in the interval (34, 35). The effective population size is always smaller than the actual population size as the effective population size exhibits the number of individuals that contribute to offspring to the descendant generation (35).

### Nucleotide sequence accession number.

Raw reads can be obtained from ENA study accession no. PRJEB11174 (http://www.ebi.ac.uk/ena/data/view/PRJEB11174) or downloaded individually from the accession number in Data Set S1 in the supplemental material.

## RESULTS

A global collection of 315 *S*. Typhimurium DT104 isolates (Europe [*n* = 235], Asia [*n* = 48], Australia [*n* = 7], North America [*n* = 18], South America [*n* = 5], and Africa [*n* = 2]) dating from 1969 to 2012 was studied. The isolates originated from animal (*n* = 197) and human (*n* = 118) sources. Seventy-five of the animal isolates were from Denmark and were selected to represent animal hosts, temporal and spatial diversity, and specific epidemiological events that had been left unexplained during the last 20 years' investigation of DT104 in Denmark. The complete details for the isolates studied can be found in Data Set S1 in the supplemental material.

Using comparative genomics, we found 4,472 core genes (out of a total of 15,098 protein clusters) from the DT104 collection meaning that, on average, about 96% of the total genes in a DT104 genome (∼4,635 genes) are common among other DT104 strains. This number is reasonable, considering the close relatedness of the DT104 strains; it is significantly higher than the 62% of genes found commonly within Salmonella enterica (36).

### Global phylogeny of *S*. Typhimurium DT104.

**FIG 1:**
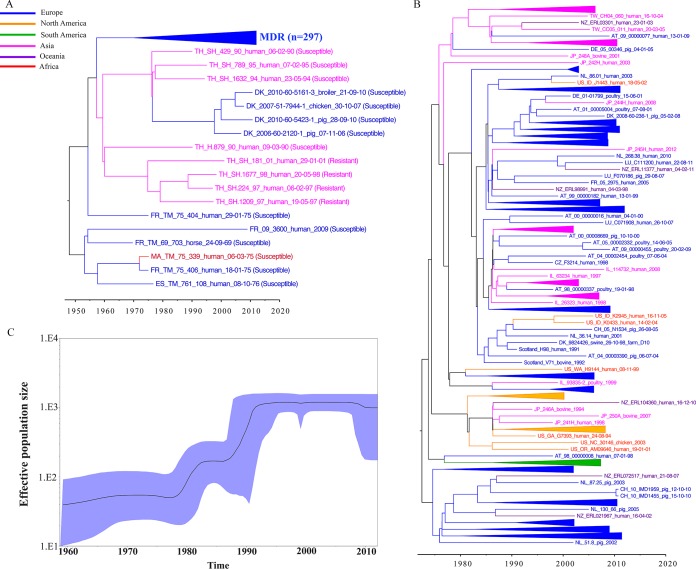
Global phylogeny of *S*. Typhimurium DT104. Bayesian-based temporal phylogenetic trees from BEAST of all DT104 (A) and subsampled MDR DT104 isolates (B). The maximum clade credibility (MCC) tree in panel A shows the most recent common ancestor of *S*. Typhimurium DT104 in ∼1948 (95% CI, 1934 to 1962) and exhibits distinct clusters between a susceptible DT104 cluster and an MDR DT104 cluster. Meanwhile, the MCC tree in panel B indicates that MDR DT104 initially emerged in ∼1972 (95% CI, 1972 to 1988). (C) The changes in effective population size over time are captured in a Bayesian skyline plot. Isolates are labeled by country of origin, isolate identification number, source, and date (day-month-year). The branches and nodes are colored according to the continent of the isolate. Country abbreviations are as follows: AT, Austria; CZ, Czech Republic; DK, Denmark; FR, France; DE, Germany; IL, Israel; JP, Japan; LU, Luxembourg; MA, Morocco; NL, The Netherlands; NZ, New Zealand; ES, Spain; CH, Switzerland; TW, Taiwan; TH, Thailand; US, United States.

The global collection of DT104 isolates was subjected to WGS, and 4,619 SNPs were identified. There were 152 significant recombination sites detected in the SNP alignment prior to the reconstruction of phylogenetic trees by RecHMM (27). Therefore, 97% (4,467/4,619) of the SNPs arose by mutation (vertical descent). We applied phylogenomic dating on the alignment of 4,467 SNPs to reconstruct the temporal and spatial phylogenetic dynamics using BEAST (Bayesian evolutionary analysis sampling trees) (29, 30). The preliminary model selection identified a combination of a Bayesian skyline model and a relaxed, uncorrelated log-normal clock as the best-supported models of population size change and molecular clock. The Bayesian maximum clade credibility (MCC) tree for all 315 DT104 isolates is shown in Fig. 1A. The mutation rate was estimated to be 2.79 × 10^−7^ substitutions/site/year, corresponding to slightly more than 1 SNP/genome/year (1.38 SNPs/genome/year). Our estimated rate of mutation coincides with the mutation rates from the previous studies of invasive *S*. Typhimurium in sub-Saharan Africa (37) and multidrug-resistant *S*. Typhimurium DT104 in different hosts (15). The most recent common ancestor was estimated to have emerged in 1948 (95% credible interval, 1934 to 1962). The tree consisted of a complex cluster of multidrug-resistant strains (MDR cluster) conferring resistance to ampicillin, chloramphenicol, streptomycin, sulfonamide, and tetracycline (ACSSuT resistance type) and subclades of susceptible and resistant isolates. The topology of this phylogenetic tree was confirmed by a maximum-likelihood tree (see Fig. S1A and B in the supplemental material). Other separated Bayesian phylogenetic trees were reconstructed from the alignment of 4,619 SNPs without removing recombination sites (see Fig. S2 in the supplemental material). The trees showed topologies similar to those of the trees free from recombination sites (Fig. 1A and B). Nonetheless, the branch lengths of the phylogenetic trees and mutation rates were different as the presence of recombination distorts the branch lengths of the phylogenetic tree (38). In addition, we constructed a maximum-likelihood tree of DT104 and 53 publicly available *S*. Typhimurium strains (see Fig. S3 in the supplemental material). The tree showed that the closest neighbors of DT104 were phage types DT12a and DT197.

The phylogenetic tree (Fig. 1A) was also analyzed according to the host (see Fig. S4 in the supplemental material). There have likely been several random transmission events among different hosts, including transmission events from human to animals and from animals to human. Transmission was also observed among different animal hosts: swine to cattle, swine to poultry, and cattle to poultry.

We also analyzed the 261 MDR isolates separately, yielding 3,621 variable sites. There were 99 significant recombination sites detected by RecHMM. Therefore, the alignment of 3,522 SNPs was subjected to Bayesian tree reconstruction using BEAST (Fig. 1B). The European isolates are disseminated throughout the tree as are the isolates from Japan, the United States, and New Zealand; in particular, the human isolates from New Zealand do not cluster together but cluster with isolates from different countries and continents (Fig. 1B), suggesting that they might be travel-related cases. This result is concordant with the report that Australia and New Zealand have had few MDR DT104 human infections, probably due to the strict regulations on importation of livestock and the fact that most human cases were travelers (4). A complete Bayesian phylogenetic tree of the 261 MDR DT104 isolates can be seen in Fig. S5 in the supplemental material.

A Bayesian skyline plot for all DT104 isolates reconstructed the demographic history of DT104 from ∼1960 (Fig. 1C). The effective population size of DT104 rose gradually until ∼1980, having acquired multidrug resistance in ∼1974, after which the population size increased sharply from 1980 to 1985 (Fig. 1C). This coincided with the initial dissemination of MDR DT104 throughout Europe, Asia, and North and South America during the 1980s (Fig. 1B). The second wave of DT104 started in ∼1990, and the population size increased dramatically. This increase may reflect the global dissemination of MDR DT104 because the timeline is consistent with the occurrence of MDR DT104 in many countries. Germany experienced an increase in DT104 at the beginning of the 1990s (39, 40). The number of DT104 human infections in the United Kingdom rose from 259 in 1990 to 4,006 in 1995 (41), while the number of DT104 infections in animals increased from 458 in 1993 to 1,513 in 1996 (7). Almost 67% of the Salmonella isolates from animals in Scotland during 1994 to 1995 were MDR DT104 (42), and a number of studies have shown that throughout the 1990s, MDR DT104 spread to other parts of the world, including the United States, the United Kingdom, and France (43–46). The trend in the skyline plot has leveled off since 1995 and gradually decreased from 2008.

The susceptible-resistant and MDR clusters differed by approximately 109 SNPs (Fig. 1A). The average SNP difference among isolates in the susceptible-resistant cluster (*n* = 18) was 103 SNPs, whereas the SNP difference among isolates in the MDR cluster, where the isolates (*n* = 297) were sampled more thoroughly, was 60 SNPs (range, 38 to 100 SNPs).

The SNP distribution across genes in DT104 was likely random with a few genes containing more than 5 SNPs (see Fig. S6 in the supplemental material). The scatter plot of the SNPs found in the susceptible and MDR strains (see Fig. S7 in the supplemental material) showed that most of the SNPs were found exclusively in some of the MDR strains and 14 SNPs were uniquely found between 62 and 74% of all MDR strains. In addition, 4 SNPs were absent in the MDR strains but present in all of the susceptible strains.

**FIG 2:**
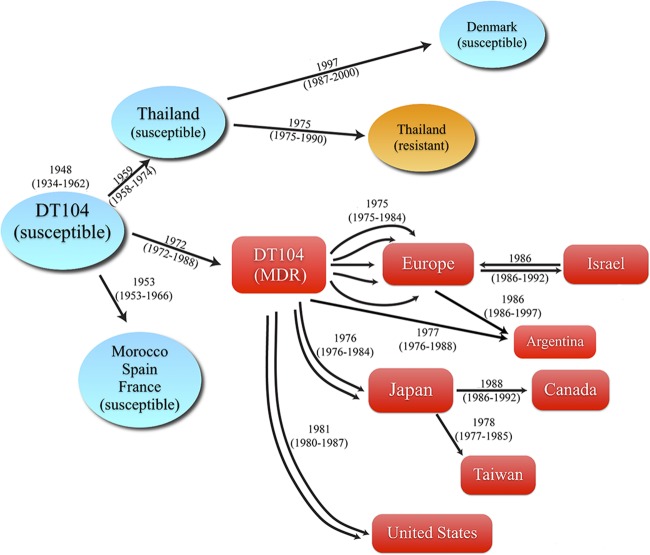
Diagram of the dissemination of *S*. Typhimurium DT104. The ages of the nodes and the divergence times of the key events from Fig. 1A and B are summarized and illustrated in this diagram. Ancestral *S*. Typhimurium DT104 initially emerged as susceptible strains in ∼1948 (95% CI, 1934 to 1962). The susceptible DT104 was estimated to acquire multidrug resistance in ∼1972 (95% CI, 1972 to 1988). The ancestral MDR DT104 spread to Europe and other continents in ∼1975 and the 1980s, respectively. The estimated times when transmissions initially occurred (in years) are presented as the median values with 95% CI in parentheses.

Based on the dates of the nodes estimated in the phylogenetic trees (Fig. 1A and B), the proposed transmissions are illustrated in Fig. 2. *S*. Typhimurium DT104 appears to have originated as a susceptible strain in 1948 (95% CI, 1934 to 1962) from an unidentified source. Susceptible strains later emerged in Morocco, Spain, and France in ∼1953 (95% CI, 1953 to 1966). In ∼1959 (95% CI, 1958 to 1974), the susceptible ancestral DT104 appeared in Thailand, where it was likely transferred onward to Denmark in ∼1997 (95% CI, 1987 to 2000). Locally in Thailand, the susceptible strains evolved resistance to streptomycin (*aadA2*) and sulfonamide (*sul1*) in ∼1975 (95% CI, 1975 to 1990).

We estimated that MDR DT104 emerged in ∼1972 (95% CI, 1972 to 1988) (Fig. 1B and 2). From an unknown source, multiple introductions of MDR DT104 occurred in Europe from ∼1975 (95% HPD, 1975 to 1984). Subsequently, further introductions to and from Israel occurred in ∼1986 (95% HPD, 1986 to 1992). Separate events transmitted MDR DT104 to Japan in ∼1976 (95% HPD, 1976 to 1984), from Japan to Taiwan in ∼1978 (95% HPD, 1977 to 1985), and from Japan to Canada in ∼1988 (95% HPD, 1986 to 1992). The transmission from Japan to Taiwan needs to be interpreted with caution, as there was only one Japanese isolate which confirmed this transmission. In addition, MDR DT104 of an unknown source initially spread to the United States in ∼1981 (95% HPD, 1980 to 1987), consistent with the report of the emergence of MDR DT104 in the United States, particularly in western states in early 1985 (45). Furthermore, it spread from Austria to Argentina in ∼1986 (95% HPD, 1986 to 1997), with an average of 81 SNP differences. MDR DT104 from an unknown source might have spread to Argentina in ∼1977 (95% HPD, 1976 to 1988).

### Dissemination of DT104 in Europe.

**FIG 3:**
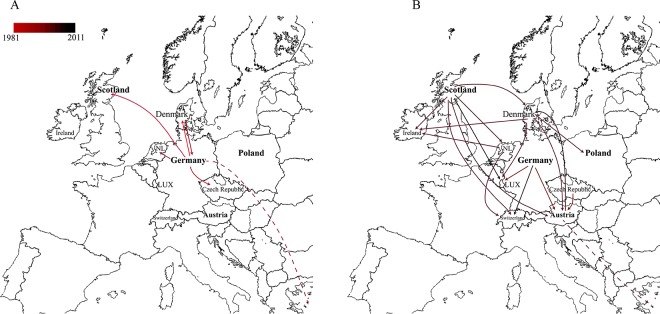
Transmissions within Europe of MDR *S*. Typhimurium DT104 from animal isolates. Discrete phylogeographic analysis of MDR DT104 during 1981 to 1990 (A) and 1990 to 2011 (B) within European countries. The locations and transmission lines were obtained from the nodes and branches in our BEAST analysis. The color gradient represents the ages of the transmission lines. Maps adapted from Wikimedia Commons (https://commons.wikimedia.org/wiki/File:Europe_blank_political_border_map.svg).

The spatial and temporal transmission of MDR DT104 isolates among animals in European countries based on discrete phylogeographic analysis using a standard continuous-time Markov chain (CTMC) is summarized and illustrated in Fig. 3. The earliest predicted dissemination (Fig. 3A) was from Germany to the Czech Republic in ∼1984 (95% CI, 1982 to 1988), from Germany to Denmark in ∼1985 (95% CI, 1982 to 1990), and from Germany to Scotland in ∼1986 (95% CI, 1984 to 1989). More recent dissemination events occurred from Denmark back to Germany in ∼1988 (95% CI, 1987 to 1994) and from Germany to The Netherlands in ∼1988 (95% CI, 1984 to 1990). In addition, Germany had transmission to Israel in ∼1988 (95% CI, 1986 to 1991). The next waves (Fig. 3B) were from The Netherlands to Ireland in ∼1992 (95% CI, 1988 to 1997) and Switzerland in ∼1993 (95% CI, 1988 to 1997). In the early 1990s, Denmark had transmission to Poland in ∼1992 (95% CI, 1988 to 1996), Austria in ∼1992 (95% CI, 1990 to 2000), Luxembourg in ∼1993 (95% CI, 1988 to 1997), and Ireland in ∼1993 (95% CI, 1989 to 2001). In the same period, Germany had an outward wave to Luxembourg in ∼1990 (95% CI, 1990 to 1998), Austria in ∼1990 (95% CI, 1988 to 1996), and Switzerland in ∼1993 (95% CI, 1990 to 1997). Scotland was another hub in the early 1990s, appearing to drive transmission to Austria in ∼1990 (95% CI, 1987 to 1991), Ireland in ∼1990 (95% CI, 1986 to 1994), The Netherlands in ∼1991 (95% CI, 1989 to 1993), Denmark in ∼1992 (95% CI, 1988 to 1994), and Switzerland in ∼1993 (95% CI, 1989 to 1995). Scotland is a net importer of food (15); 58% of all red meat and 38% of raw beef are non-Scottish in origin (16). Austria also had transmission back to Denmark in ∼1998 (95% CI, 1990 to 1999) and had a phylogenetically linked wave to Israel in 1992 (95% CI, 1989 to 1994) via isolates from poultry. The most recent predicted transmission was from Scotland to Luxembourg in ∼2000 (95% CI, 1998 to 2005).

### Local phylogeny of *S*. Typhimurium DT104.

**FIG 4:**
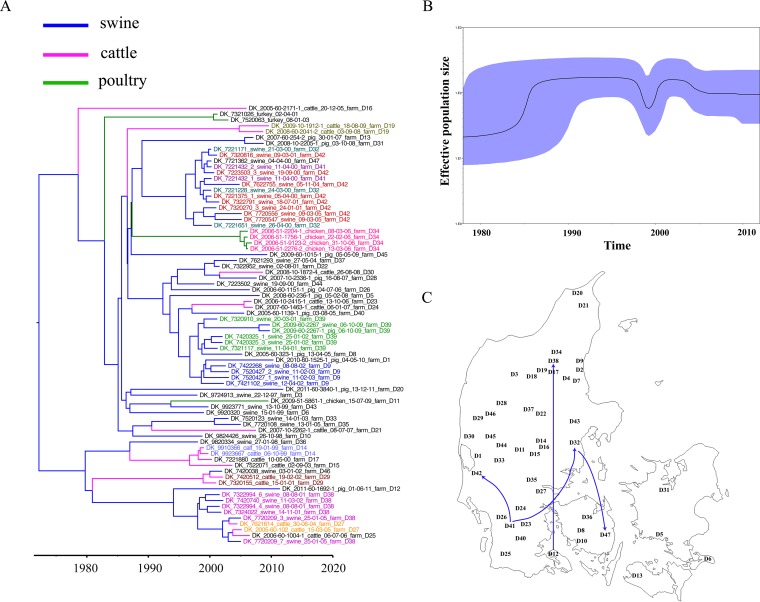
Local phylogeny of MDR *S*. Typhimurium DT104 isolates in Denmark. (A) Bayesian phylogenetic tree of 75 Danish MDR DT104 isolates showing that the most recent common ancestor is estimated to have emerged in ∼1972 (95% CI, 1961 to 1982). The tree is further divided into two major clusters in ∼1979 (95% CI, 1969 to 1987) and ∼1980 (95% CI, 1970 to 1988). The farm numbers are noted at the ends of the node names. The nodes are colored according to the farm of origin. Strains originating from the same farm are labeled the same color except that black is used for a single isolate originating from a single farm. Colored branches show animal sources. (B) Bayesian skyline plot of changes in population size of Danish MDR DT104 over time. (C) Geographic diffusion across different farms based on discrete phylogeographic analysis for the confirmed-farm contacts. The complete geospatial transmission is provided in Video S1 in the supplemental material. Map adapted from d-maps.com (http://www.d-maps.com/m/europa/danemark/danemark42.gif).

Seventy-five MDR *S*. Typhimurium DT104 isolates sampled from 1997 to 2011, originating from several farms in Denmark, were part of the larger collection, among which 755 SNPs were identified. A total of 108 recombination sites were identified. The sequence alignments of 647 SNPs separating these isolates were analyzed using BEAST. The Bayesian phylogenetic tree (Fig. 4A) estimated a mutation rate of 2.50 × 10^−7^ substitutions/site/year or 1.23 SNPs/genome/year. The most recent common ancestor was predicted to have emerged in ∼1972 (95% CI, 1961 to 1982). The tree was initially divided into two complex clusters and subsequently branched off into many lineages, indicating multiple introductions of MDR DT104 to different farms in Denmark. The topology of the Bayesian tree was concordant with the maximum-likelihood tree of Danish MDR DT104 (see Fig. S8 in the supplemental material).

Several isolates were sampled from the same farms. Most of those isolates clustered phylogenetically according to their farms. Isolates from four different farms namely D32, D41, D42, and D47 were mixed within the same lineage. This is consistent with epidemiological information reporting physical contact among those four farms, thus confirming the ability of WGS to detect very local transmission dynamics. Considering all the farm-associated isolates, there appear to have been several transmission events between swine and cattle (Fig. 4A), whereas isolates from poultry clustered together. This indicates free transmission between cattle and swine, but a more closed spread among poultry isolates and is consistent with the analysis of proliferation of the infection in various species, which suggested that DT104 strains spread from cattle to pigs and humans (7, 47), unlike the global transmission events of DT104, which are random and not specific to the host (see Fig. S4 in the supplemental material).

The relationship between the population structure and time (Fig. 4B) showed that the effective population size of MDR DT104 in Denmark rose slowly until ∼1984 and then increased sharply from ∼1984 to ∼1987. Subsequently, the population was stable until ∼1998, and it declined dramatically during ∼1999 to ∼2000, when an intensive eradication program was attempted in Denmark (48). Following the abandonment of the eradication program, the population size increased in ∼2001 and has decreased slightly since ∼2004. We carried out different Bayesian skyline plots based on different animal and human sources (see Fig. S9 in the supplemental material). The pattern of sharp decline during 2000 appears to be restricted to swine isolates and was not apparent among isolates from cattle, poultry, and humans. In fact, 69% of the Danish isolates were from swine. Thus, we conclude that the decline of the population size in 2000 was related to a decrease in swine infection/colonization.

Discrete phylogeographic analysis indicated several transmission events between farms in Denmark. The complete transmission events can be found in Video S1 in the supplemental material, which is a video recorded from the KML file (KLM is a file format used to display geographic data in an Earth browser such as Google Earth or Google Maps). The average SNP distances between the isolates from the farms ranged from 3 to 100 SNPs. We have four confirmed physical contacts between the farms. Those contacts were concordant with the phylogeographic links shown in Fig. 4C. The contacts between farms D12 and D38 and D41 and D42 were direct relationships with 30 and 7 SNP differences, respectively, whereas the contacts from farms D32 and D42 and D42 and D47 were indirect contacts corresponding to 10 and 8 SNP distances, respectively. Interestingly, data from one farm (D10) where eradication was presumably unsuccessful showed that isolates found posteradication were not the same lineage as the isolates found prior to eradication.

### Salmonella genomic island 1 and resistance genes.

**FIG 5:**
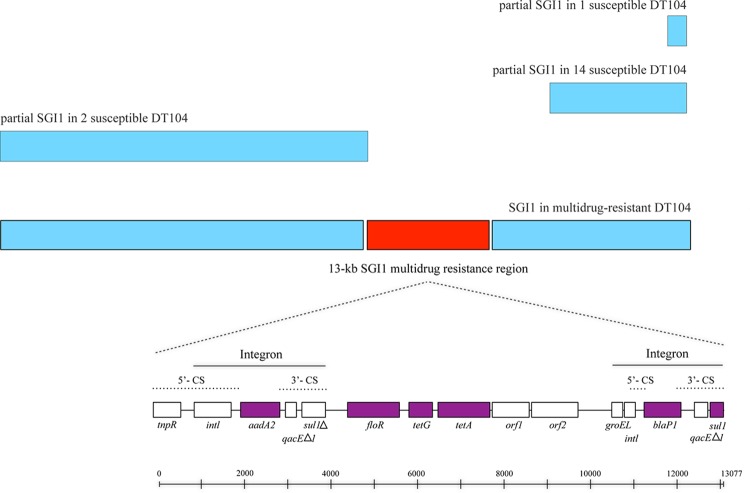
Structure of SGI1 in susceptible DT104 and SGI1 containing a 13-kb MDR region in MDR DT104 isolates. The gene organization of the MDR region of *S*. Typhimurium DT104 is illustrated. The antimicrobial resistance gene cassettes are colored purple. The *aadA2* gene cassette confers resistance to streptomycin and spectinomycin. The *floR* conferring resistance to chloramphenicol and florfenicol and the *tet*(G) and *tetA* conferring resistance to tetracycline reside between the two integron-derived regions. The *blaP1* gene cassette confers resistance to ampicillin. A complete *sul1* sulfonamide resistance gene cassette is located in the 3′-CS on the right.

All of the isolates in the susceptible-resistant clusters contained small fragments or partial sequences of the 43-kb Salmonella genomic island 1 (SGI1) (GenBank accession number AF261825) (49, 50), but none of them harbored the 13-kb SGI1 multidrug resistance region (51) (Fig. 5). The phylogenetic tree based on the SNPs of SGI1 of all of the DT104 isolates and other Salmonella and Proteus mirabilis genomes that carry SGI1 are shown in Fig. S10 in the supplemental material. The tree showed that the SGI1 sequences of the DT104 isolates were very similar, and they were similar to SGI1 sequences from other Salmonella and P. mirabilis genomes. The SGI1 tree showed a topology similar to that of the tree of the entire genome of DT104 (Fig. 1A; see also Fig. S1 in the supplemental material) and also showed that the four Thai resistant isolates were distinct from the other resistant strains. The gene organizations of the antimicrobial resistance genes in the 13-kb region are shown in Fig. 5. The maximum-likelihood trees of each resistance gene [*aadA2*, *floR*, *tet*(G), *blaP1*, and *sul1*] from the DT104 isolates and other bacterial genomes are shown Fig. S11A to E in the supplemental material. The trees show that the sequences of the *floR* and *tet*(G) genes among the DT104 isolates are similar and formed a cluster distinct from those of the same genes from other bacterial species, whereas there was more variation for the *aadA2*, *blaP1*, and *sul1* genes.

## DISCUSSION

### Global epidemiology.

*S*. Typhimurium DT104 has gained intensive global interest due to its rapid intercontinental dissemination, the chromosomal location of multiple resistance genes, and its capacity to promptly acquire additional resistance traits (4). Our analysis of a global collection of DT104 isolates suggests that the most recent common ancestor of *S*. Typhimurium DT104 emerged in ∼1948 (95% CI, 1934 to 1962) in an antimicrobial-susceptible form (Fig. 1A) from an unidentified reservoir. The earliest reports on susceptible DT104 strains isolated from human infections appeared in 1960s in the United Kingdom (6). However, most, if not all, nontyphoidal Salmonella serovars have their natural reservoir in animals and only occasionally infect humans. Thus, susceptible DT104 may easily have spread for several years in an animal reservoir before the first infections occurred in humans. Interestingly, our results suggest that the ancestral susceptible DT104 spread to Thailand in ∼1959 (95% CI, 1958 to 1974) and later acquired resistance locally in ∼1975 (95% CI, 1975 to 1990) in Thailand (Fig. 1A and 2). It has previously been assumed that these resistant isolates (ACSSuT) emerged from an MDR strain (ampicillin, chloramphenicol, streptomycin, sulfonamides, tetracyclines, trimethoprim and ciprofloxacin [ACSSuTTmCp]) that lost some of its resistance genes (6). However, our study refutes this hypothesis.

Our results suggest that the earliest multidrug-resistant DT104 arose independently in ∼1972 (95% CI, 1972 to 1988) from an unknown source (Fig. 1B and 2). The first observations of MDR DT104 in humans were in Hong Kong in the late 1970s, and the first observation in seagulls and cattle were in the United Kingdom in 1984 (6, 39, 52), where it was thought to have originated from gulls and exotic birds imported from Indonesia and Hong Kong (6). An Asian origin has also been suggested in other previous studies, where it was indicated that the resistance determinants of MDR DT104 strains may have emerged among bacteria in aquaculture (most farmed shrimp are produced in Asia, particularly in China and Thailand) and were subsequently horizontally transferred to *S*. Typhimurium DT104 (53). It might be that aquaculture bacteria caused the emergence of Thai resistant DT104. Our study refutes this hypothesis. Based on our results, a European origin of MDR DT104 seems much more likely. Accordingly, the isolates from Thailand are not involved in the MDR DT104 cluster and MDR DT104 did not emerge in the countries from which we have isolates prior to 1980.

The phylogenomic tree based on the host association (see Fig. S4 in the supplemental material) indicated several host switch events between different animal species: from animals to humans and also likely from humans to animals. The conclusions on the host switches have to be interpreted with care since not all host species are represented for all geographic regions (e.g., no human isolates from Denmark and no animal isolates from Thailand). The zoonotic nature of DT104 is well documented (4, 54–56), but this study documents the ubiquitous nature of the bacterium and the fact that the global emergence has been one of shared epidemics with multiple transmission events between countries and animal hosts and likely also events of human to animal transmission. Nonetheless, the predictive powers of DT104 transmission and host preference were obstructed by a limited number of strains and software to infer phylogeny and evolution.

The Bayesian phylogenetic tree revealed that the susceptible and MDR clusters differed by 109 SNPs, indicating that these two clusters are diverse. The 18 isolates within the susceptible cluster had 103 SNP differences, while there were 60 SNP distances within the MDR cluster (*n* = 297), suggesting that the MDR strains were more genetically uniform. From the sequence comparisons, we found that partial or complete SGI1 was present in all isolates and the main variation was the presence or absence of the different resistance gene cassettes.

SGI1 is a 43-kb genomic island containing 44 open reading frames (ORFs). The antimicrobial resistance gene cassettes have resided in a 13-kb segment of the SGI1, namely the MDR region (49, 57). SGI1 is non-self-transmissible, but it is mobilizable by the conjugative machinery of an IncA/C plasmid (50). Therefore, it is considered an integrative mobilizable element (58).

The 13-kb MDR region contains class 1 integrons with the presence of a 5′-conserved segment (5′-CS), consisting of the insertion sequence IS*6100* (59) (Fig. 5). Further, the MDR region is surrounded by 5-bp direct repeats, suggesting that it integrated into the SGI1 by a transposition event (59, 60). The GC content of SGI1 is 49.17% compared to 58.7% for the MDR region within SGI1 (57), suggesting a potentially horizontal transfer of the MDR region into SGI1. Another indication for horizontal transfer of the antibiotic resistance gene cluster is that this cluster is present in another serovar, S. enterica serovar Agona (61). In addition, the DT104 resistance genes can be transduced by the P22-like phage ES18 and by phage PDT17, which are produced by all DT104 isolates so far encountered (62). Moreover, a phylogenetic analysis of SGI1 (see Fig. S10 in the supplemental material), excluding resistance genes, from DT104 and other bacterial species showed that the islands were highly similar. These support the observations that SGI1, without the resistance genes, is intrinsic to DT104 and that the resistance genes were acquired later. The phylogenetic analysis also indicates that SGI1 from other Salmonella serovars and P. mirabilis might have been acquired mainly from DT104. Our results challenge the hypothesis that MDR DT104 emerged by acquiring an entire SGI1 with an MDR region (63) or emerged from an MDR strain (ACSSuT) that lost some of its resistance genes (6).

More phylogenetic variation was observed for the *aadA*, *blaP1*, and *sul1* genes (see Fig. S11A to E in the supplemental material). This suggests that these genes have either been acquired on a number of occasions or on a higher frequency of evolution of recombination. Both *floR* and *tet*(G) formed a group separated from those of other bacterial species or Salmonella serovars. Even though the number of sequences from other species was low, this suggests that these two genes have only been acquired once into MDR DT104. In addition, 14 SNPs were uniquely found among 62 to 74% of all MDR strains. These SNPs might be other factors contributing to the emergence of the MDR DT104.

### Local epidemiology.

The phylogenomic analysis was able to cluster isolates from the same herd and to cluster isolates from different confirmed contact farms, suggesting that WGS is highly useful for reconstructing local epidemiological dynamics across animal herds.

The reconstructed changes in effective population sizes over time also provided an interesting insight in that there was a sharp decline in the population size of swine-associated MDR DT104 during ∼1999 to ∼2000 and a recovery in the population size to the same state prior to decreasing since ∼2001. The decrease of swine MDR DT104 is evidence of the success of the eradication program in 1996 to 2000 implemented by the Federation of Danish Pig Producers and Slaughterhouses in collaboration with the Danish Veterinary Service and the Danish Veterinary Laboratory. The program aimed to eradicate MDR DT104 from infected pig herds. The methods used included the depopulation of pig herds and the cleaning and disinfection of buildings before repopulation with pigs free of DT104 (48). In 2000, the program was stopped due to no evidence of success, but if WGS had been available at that time, such evidence would have been found.

In conclusion, this study charts the timeline of global and local dissemination of *S*. Typhimurium DT104 and the evolution of antimicrobial-susceptible strains to multidrug-resistant DT104 strains through horizontal transfer of the 13-kb SGI1 MDR region. The results are consistent with the historical emergence of MDR DT104 since it was first observed in 1984. Moreover, the results revealed by WGS confirm the local epidemiology of DT104 and the efficiency of the eradication program in Denmark. The inferred transmission routes and demographic history might suggest some potential monitoring and strategies for further prevention and control of similar successful clones.

## Supplementary Material

Supplemental material
