# Supplementary material for: Global Genomic Epidemiology of Salmonella enterica Serovar Typhimurium DT104
Source: Appl Environ Microbiol. 2016 Apr 4;82(8):2516–26. doi: 10.1128/AEM.03821-15 (PMC4959494; doi:10.1128/AEM.03821-15)
Supplement: Supplemental material [file supp_82_8_2516__index.html]

Global Genomic Epidemiology of Salmonella enterica Serovar Typhimurium DT104 — Supplemental material 

# Global Genomic Epidemiology of Salmonella enterica Serovar Typhimurium DT104

## Supplemental material

- Supplemental file 1 -

  Maximum likelihood trees of all DT104 isolates (Fig. S1), Bayesian phylogenetic trees with recombinations (Fig. S2), maximum likelihood tree of 315 DT104 and 53 publicly available *S.* Typhimurium isolates from NCBI (Fig. S3), Bayesian phylogenetic tree of 315 DT104 isolates showing host association (Fig. S4), complete Bayesian phylogenetic tree of 261 MDR DT104 isolates (Fig. S5), distribution of SNPs across genes in DT104 (Fig. S6), scatter plot of SNPs found in susceptible and MDR strains (Fig. S7), maximum likelihood tree of MDR Danish strains (Fig. S8), individual Bayesian skyline plots for different sources (Fig. S9), SNP tree of SGI1 from DT104 isolates and other bacterial species (Fig. S10), and maximum likelihood tree of genes from DT104 isolates and other bacterial species (Fig. S11).

  PDF, 4.6M
- Supplemental file 2 -

  The complete transmission events of MDR DT104 between farms in Denmark (recorded from the original KML file that is a file format used to display geographic data in an Earth browser such as Google Earth/Google Maps) (Video S1).

  MOV, 8.0M
- Supplemental file 3 -

  Epidemiological and genomic information on *S.* Typhimurium DT104 in this study (Data Set S1).

  XLSX, 95K
