# Supplementary material for: Global Genomic Epidemiology of Salmonella enterica Serovar Typhimurium DT104
Source: Appl Environ Microbiol. 2016 Apr 4;82(8):2516–26. doi: 10.1128/AEM.03821-15 (PMC4959494; doi:10.1128/AEM.03821-15)
Supplement: Supplemental material [file AEM.03821-15_zam999117084so1.pdf]

Supporting Information

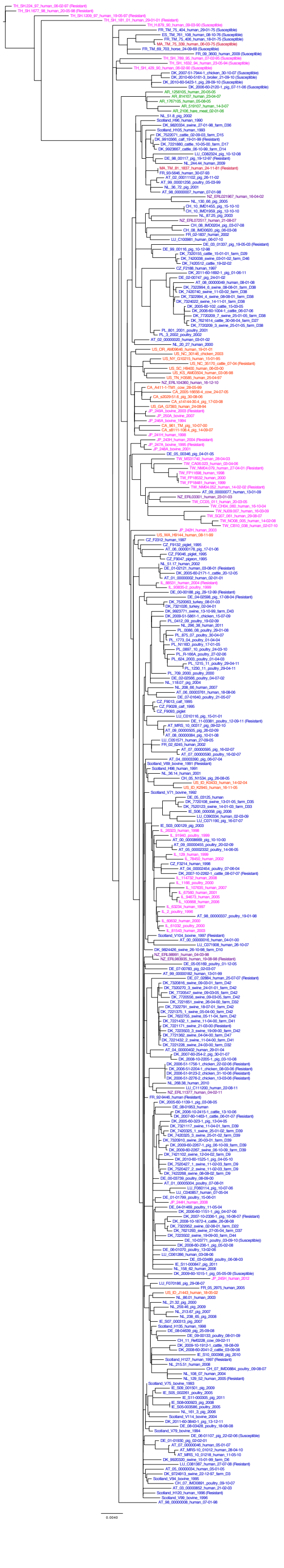

Fig. S1 (A) Maximum likelihood tree (rectangular tree layout) of all DT104 (susceptible, resistant and MDR) the isolates without labelling are MDR strains.

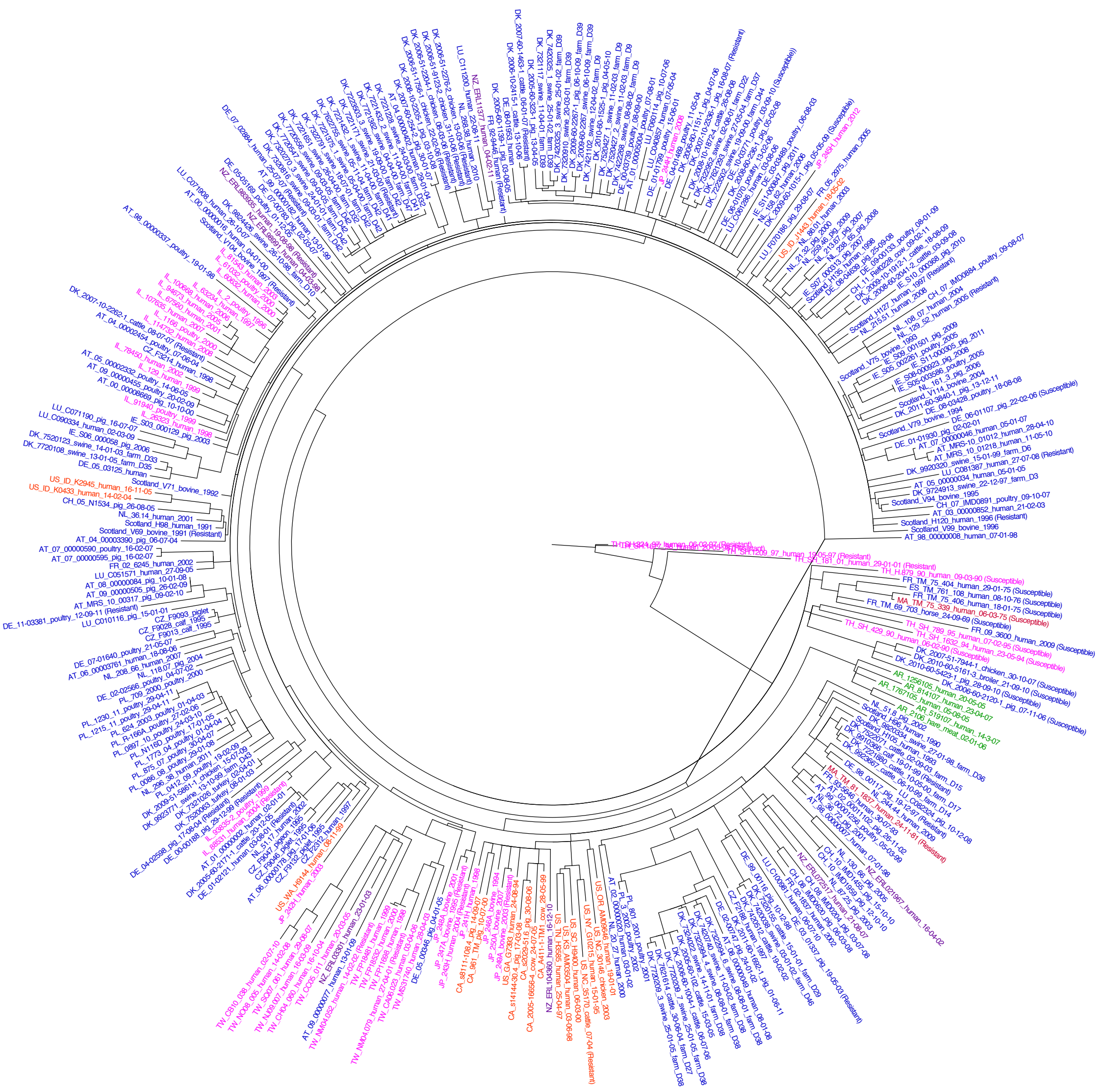

Fig. S1 (B) Maximum likelihood tree (circle tree layout) of all DT104 (susceptible, resistant and MDR) the isolates without labelling are MDR strains.

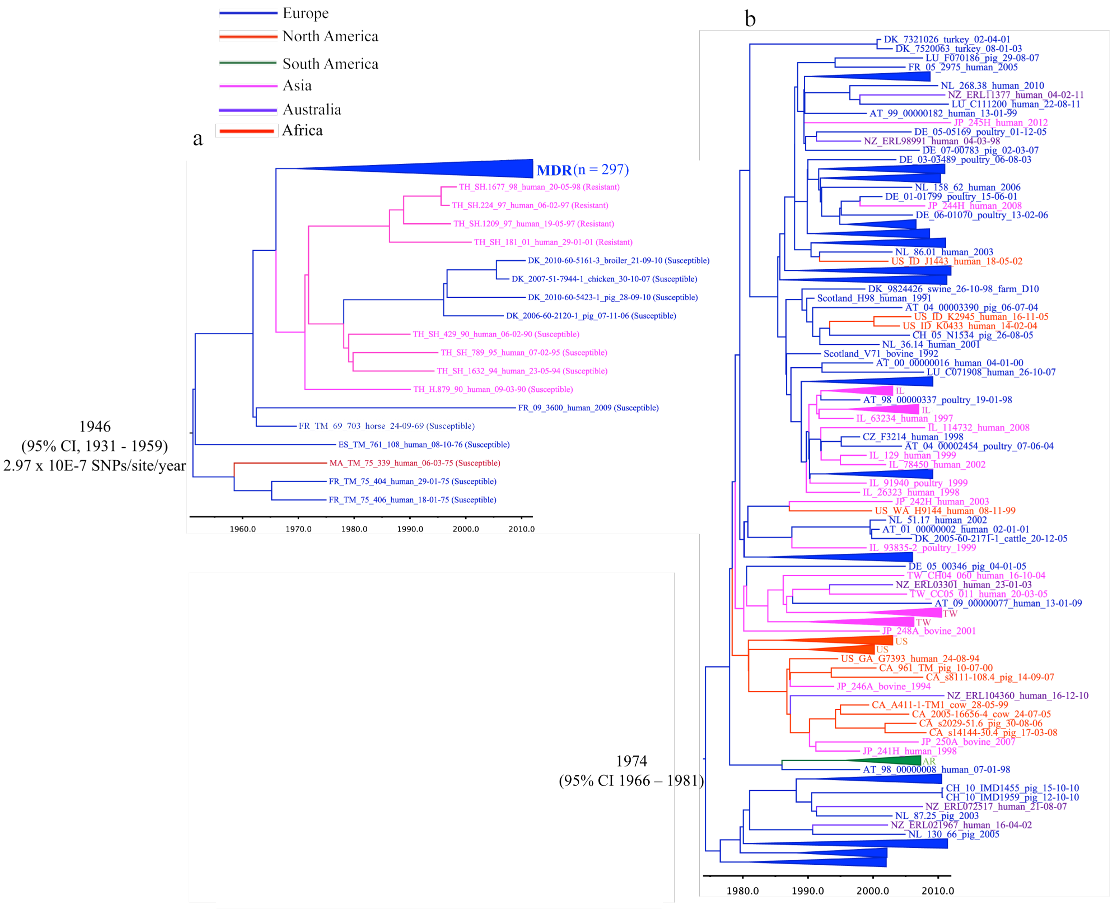

Fig. S2 Bayesian phylogenetic trees with recombinations

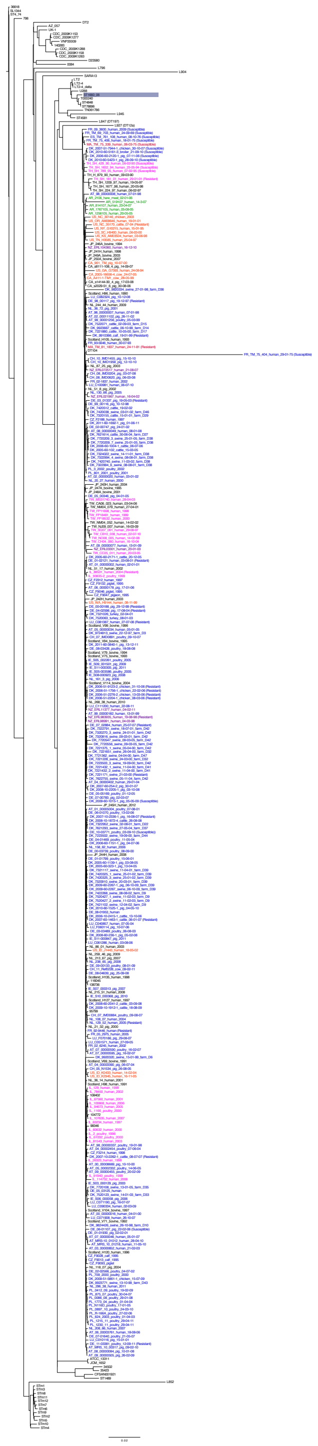

Fig. S3 Maximum likelihood tree of 315 DT104 and 53 publicly available *S*. Typhimurium isolates from NCBI

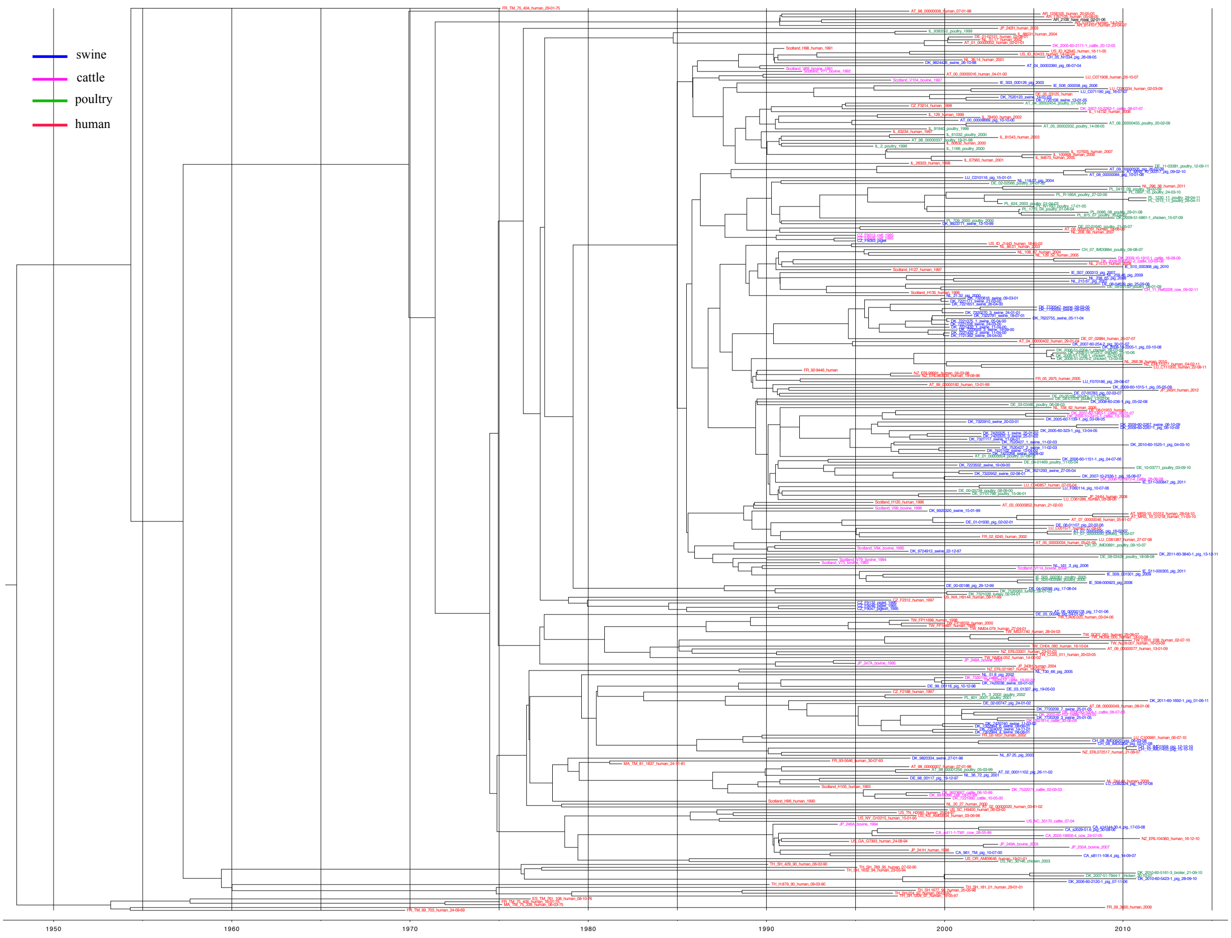

Fig. S4A

Fig. S4 (A) Complete Bayesian phylogenetic tree (rectangular tree layout) of 315 DT104 showing host association.

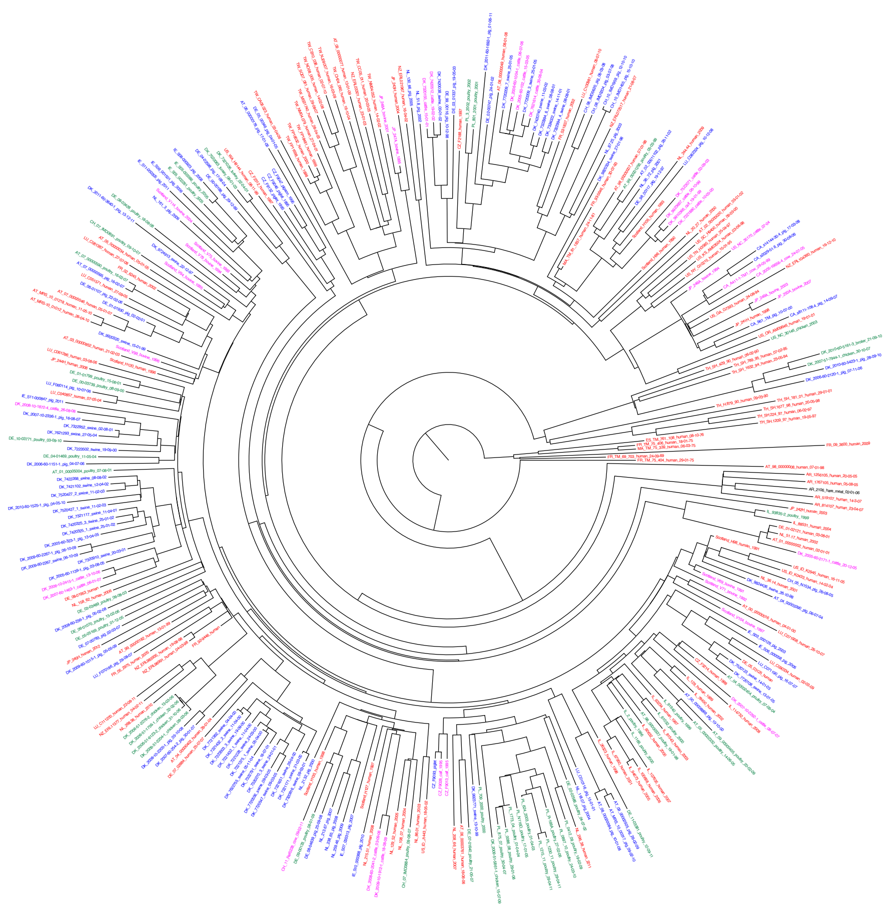

Fig. S4 (B) Complete Bayesian phylogenetic tree (circular tree layout) of 315 DT104 showing host association.

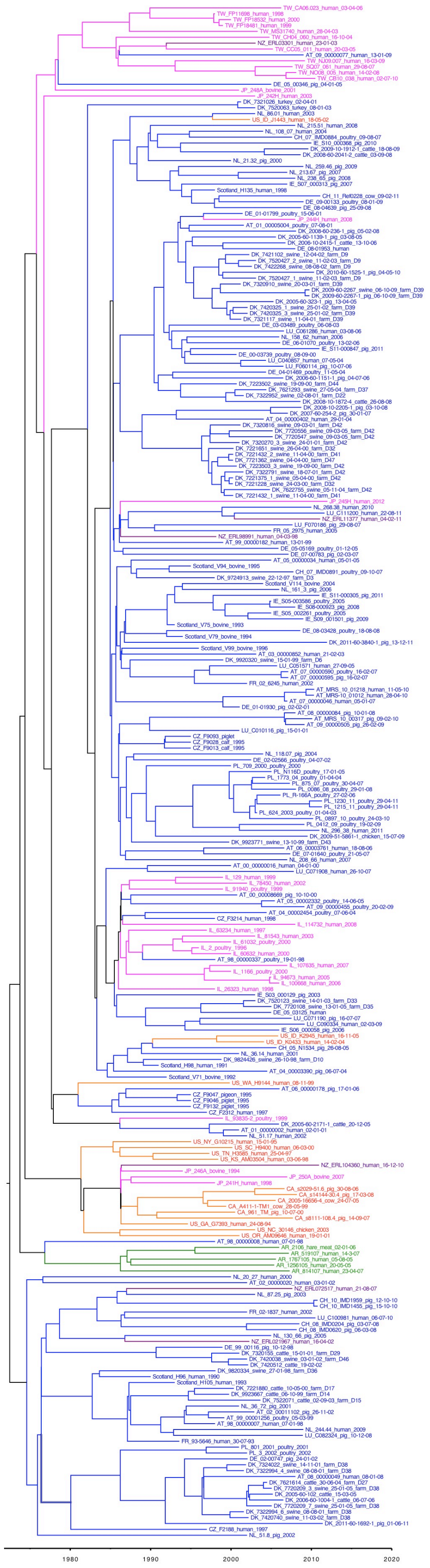

Fig. S5 Complete Bayesian phylogenetic tree of 261 MDR DT104

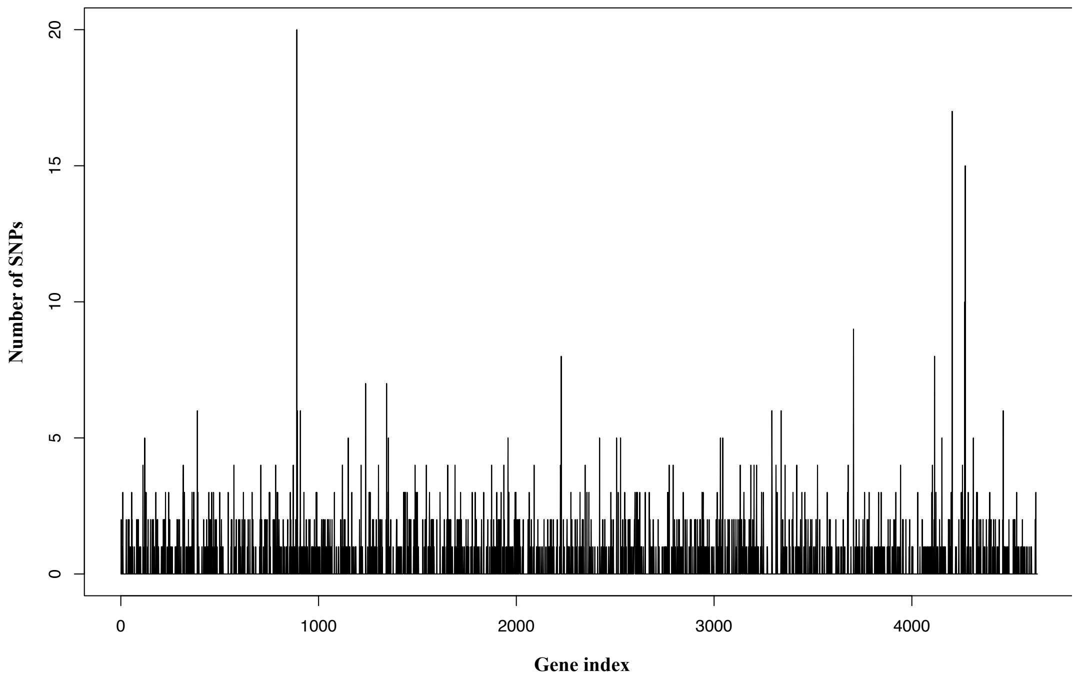

Fig. S6 Distribution of SNPs across genes in DT104

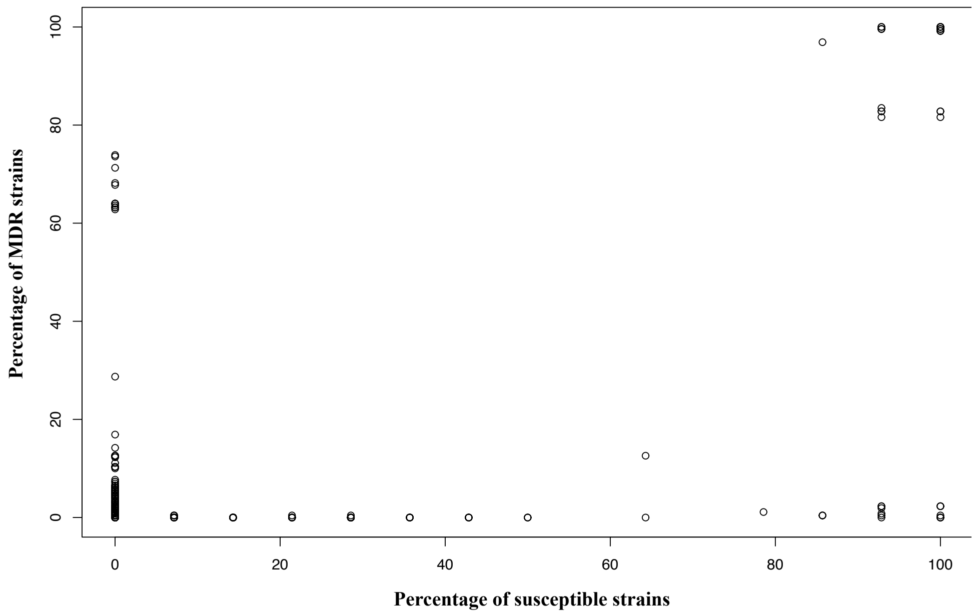

Fig. S7 Scatter plot of SNPs found in susceptible and MDR strains

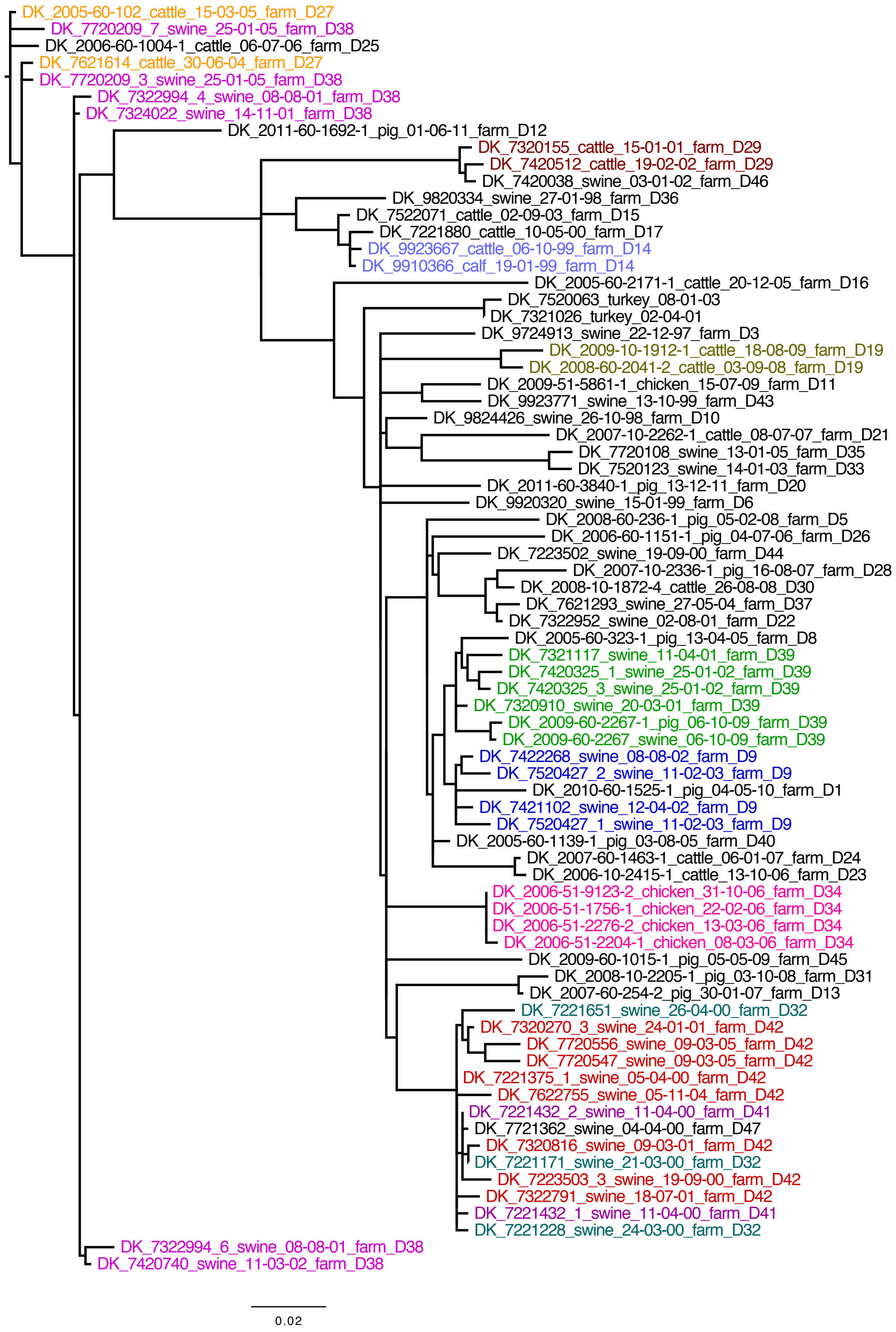

Fig. S8 Maximum likelihood tree of MDR Danish strains

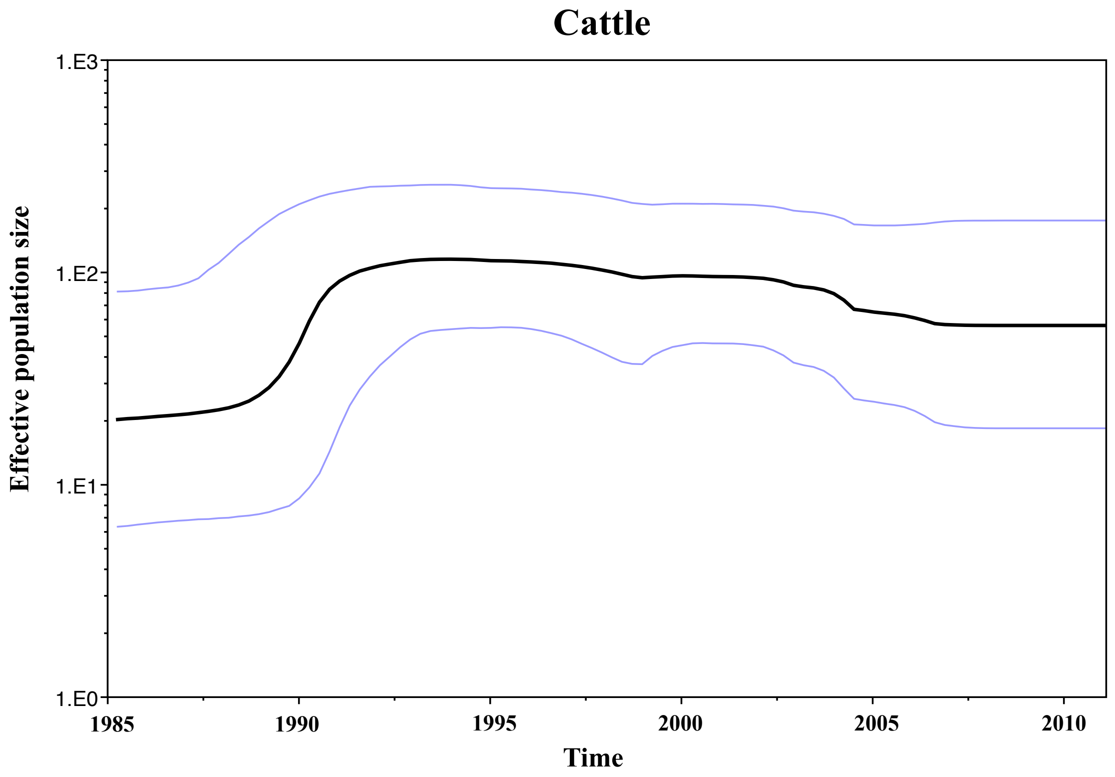

Fig. S9 (A) Individual Bayesian skyline plots for different sources; cattle

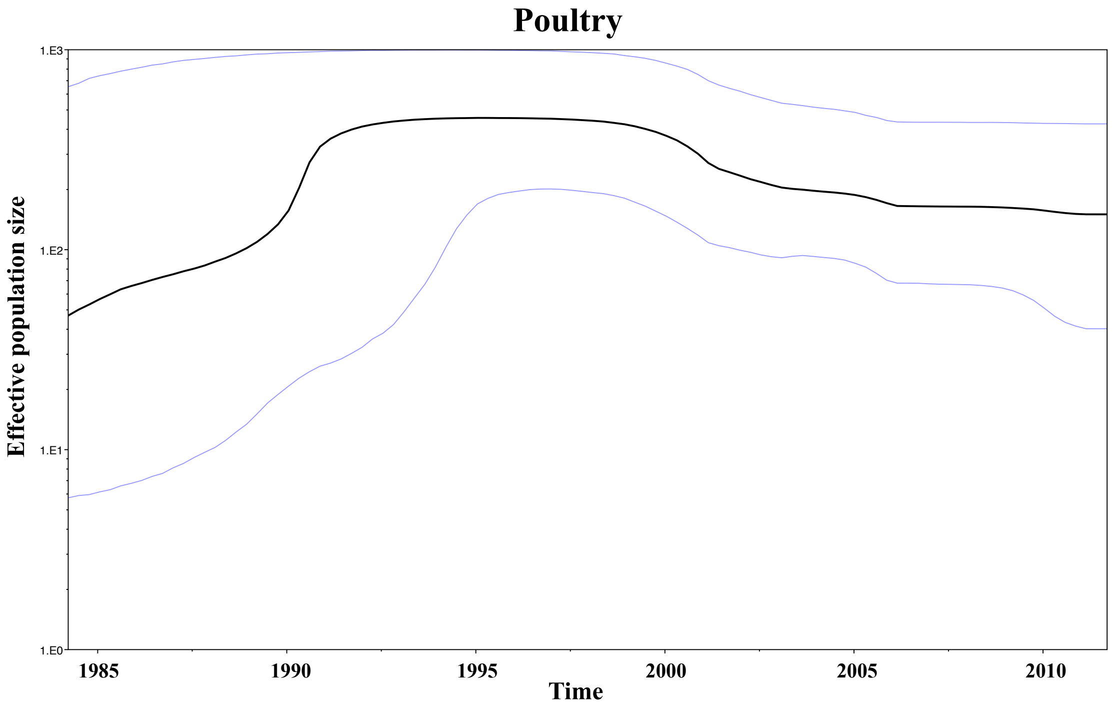

Fig. S9 (B) Individual Bayesian skyline plots for different sources; poultry

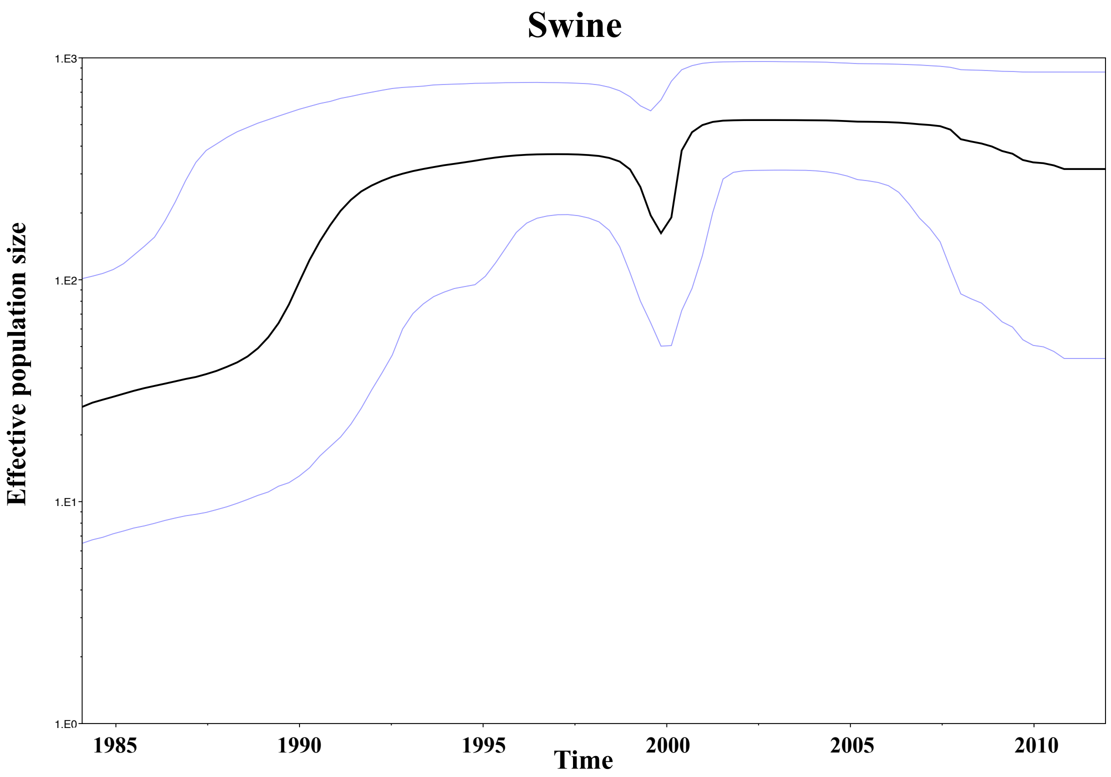

Fig. S9 (C) Individual Bayesian skyline plots for different sources; swine

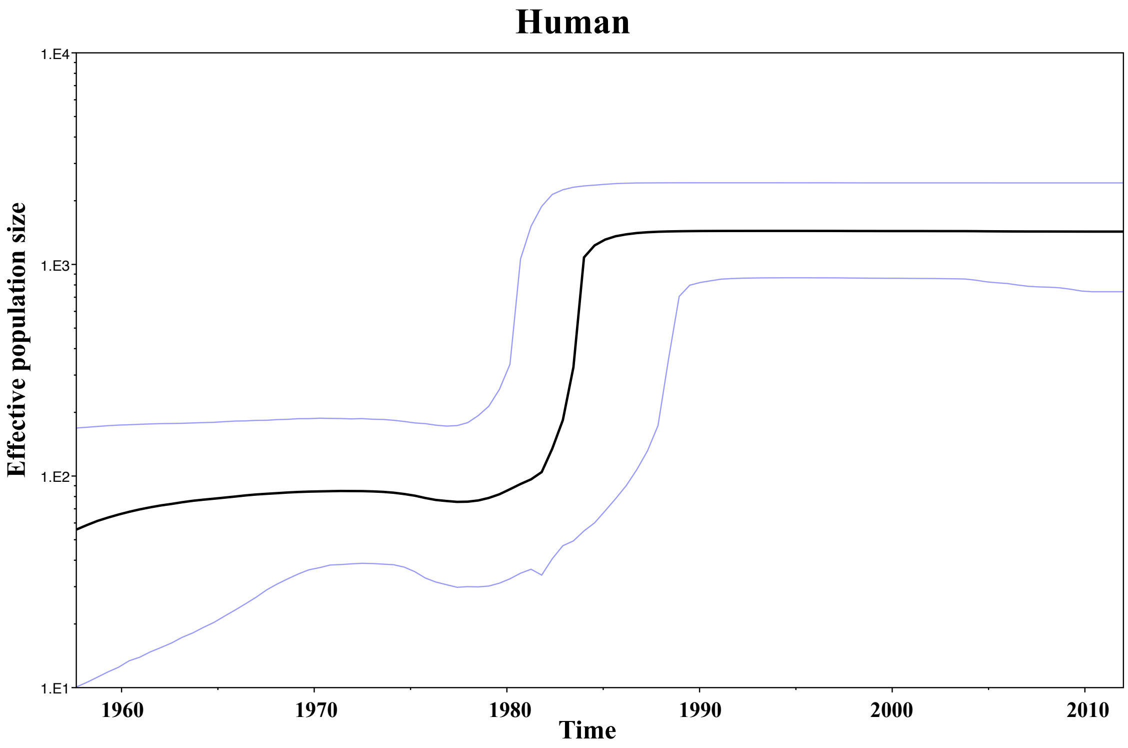

Fig. S9 (D) Individual Bayesian skyline plots for different sources; human

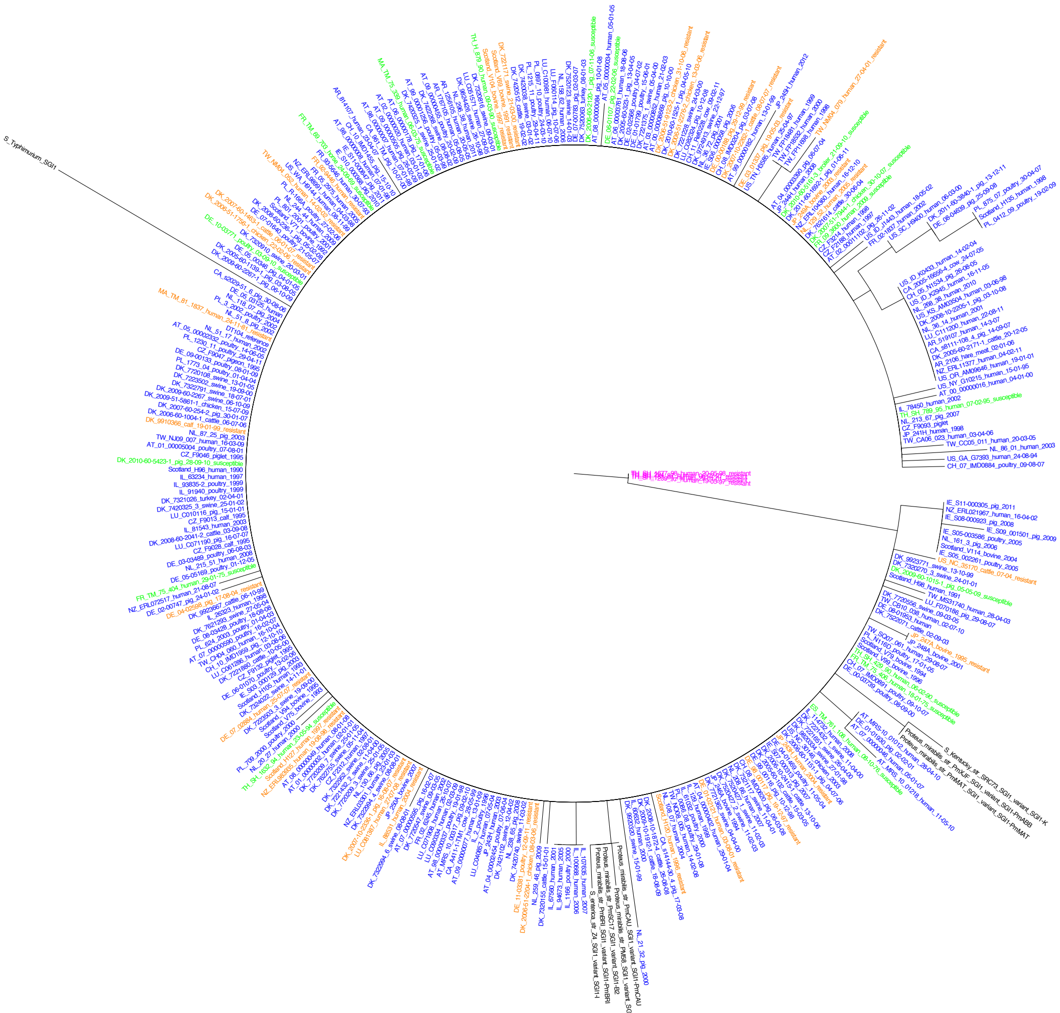

Fig. S10 SNP tree of SGI1 from DT104 isolates and other bacterial species. DT104 isolates with green label are susceptible strains, with pink label are resistant strains from susceptible cluster, with orange label are resistant strains from MDR cluster, with blue label are MDR strains. Black labels are other bacterial genomes.

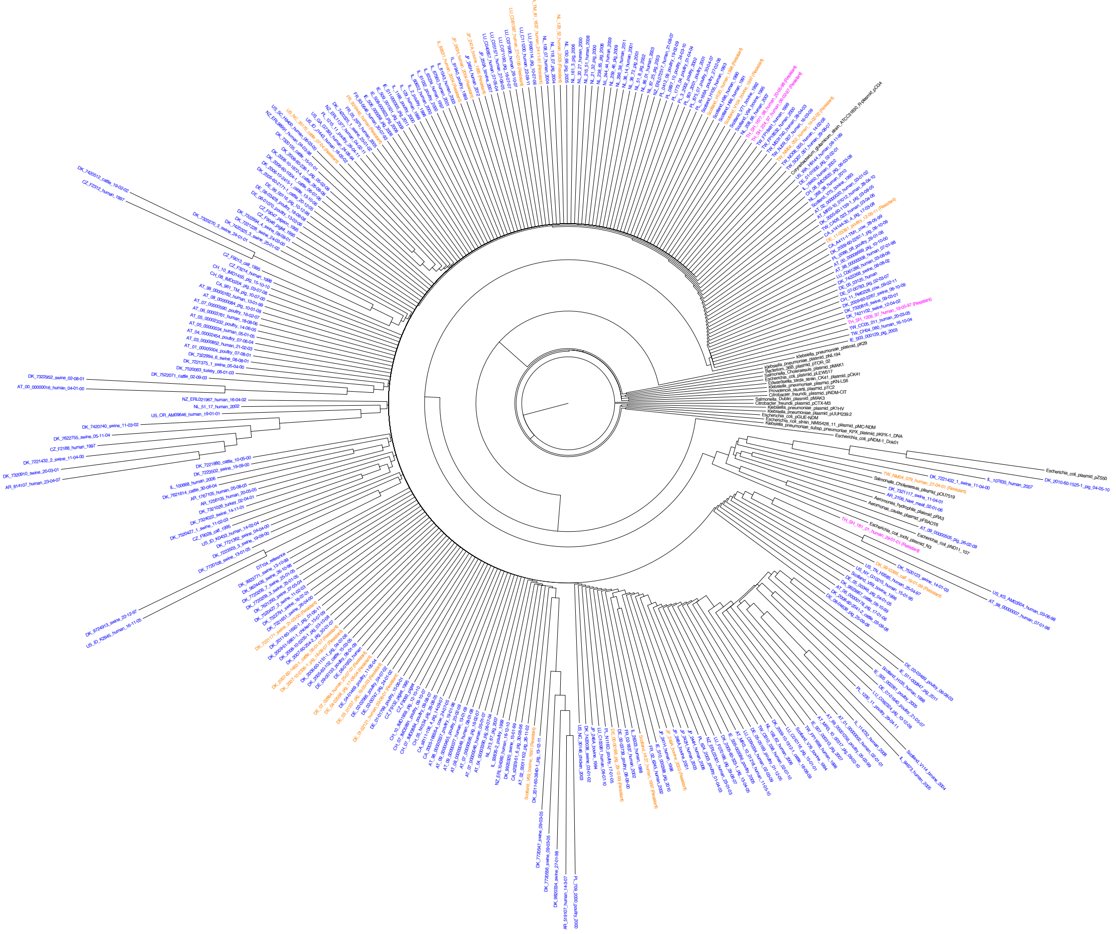

Fig. S11 (A) Maximum likelihood tree of *aadA2* genes from DT104 isolates and other bacterial species. DT104 isolates with pink label are resistant strains from susceptible cluster, with orange label are resistant strains from MDR cluster, with blue label are MDR strains. Black labels are other bacterial genomes.

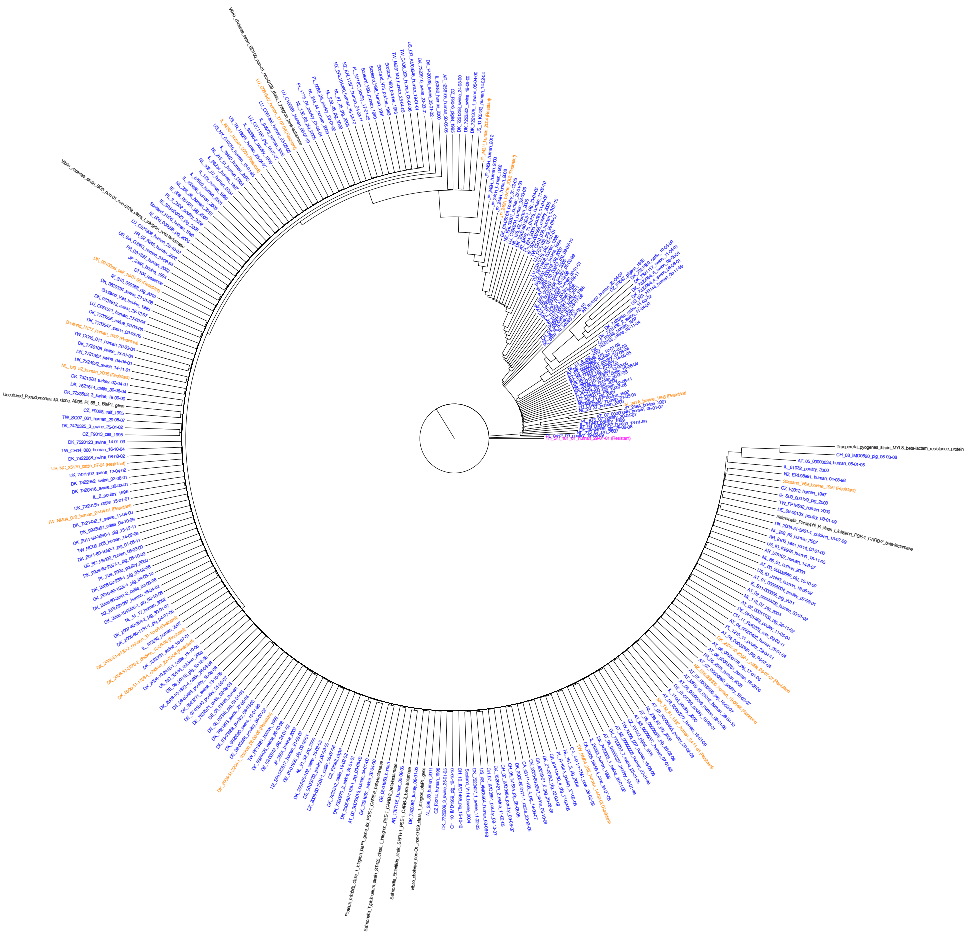

Fig. S11 (B) Maximum likelihood tree of *blaP1* genes from DT104 isolates and other bacterial species. DT104 isolates with pink label are resistant strains from susceptible cluster, with orange label are resistant strains from MDR cluster, with blue label are MDR strains. Black labels are other bacterial genomes.

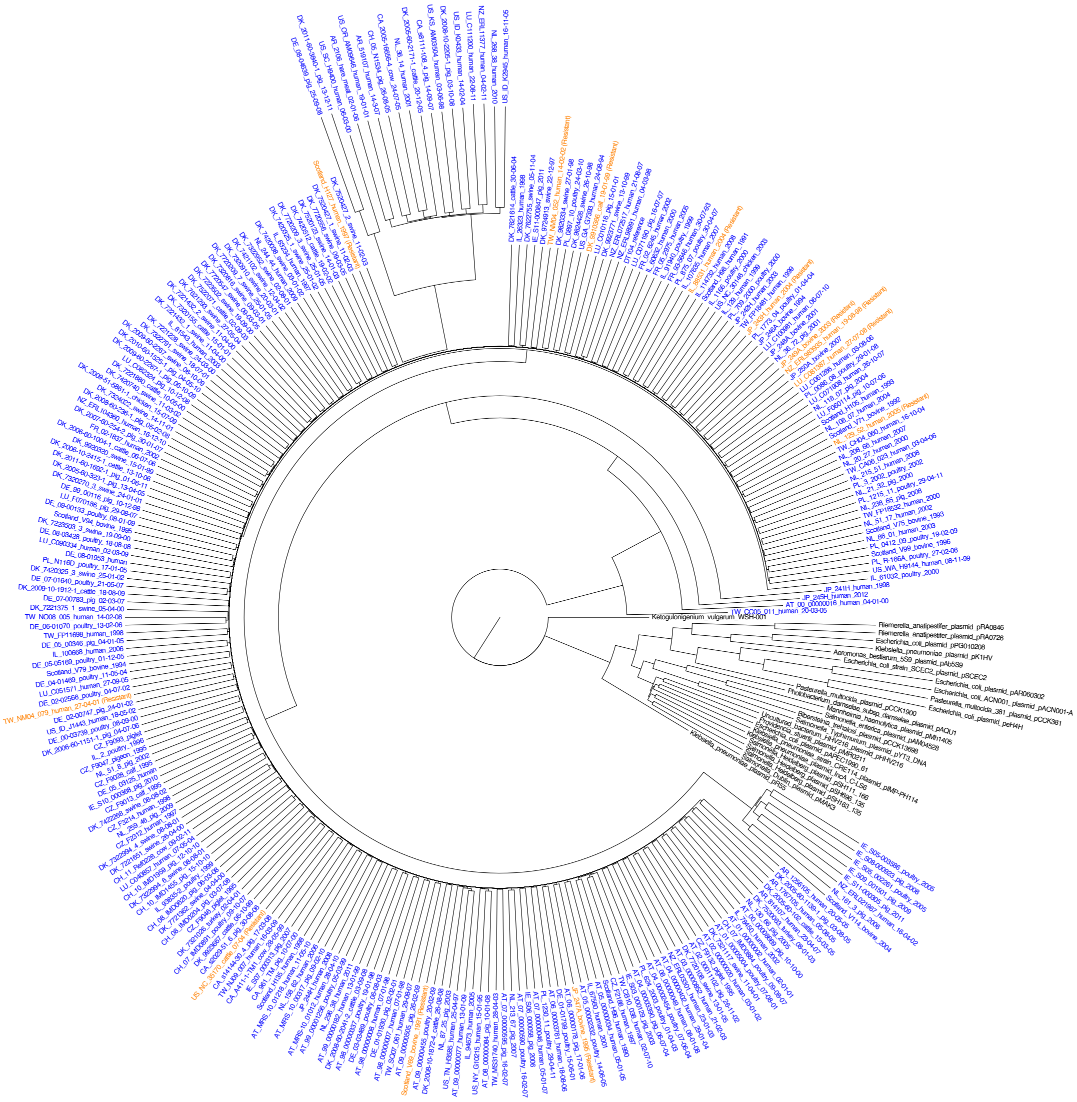

Fig. S11 (C) Maximum likelihood tree of *floR* genes from DT104 isolates and other bacterial species. DT104 isolates with pink label are resistant strains from susceptible cluster, with orange label are resistant strains from MDR cluster, with blue label are MDR strains. Black labels are other bacterial genomes.

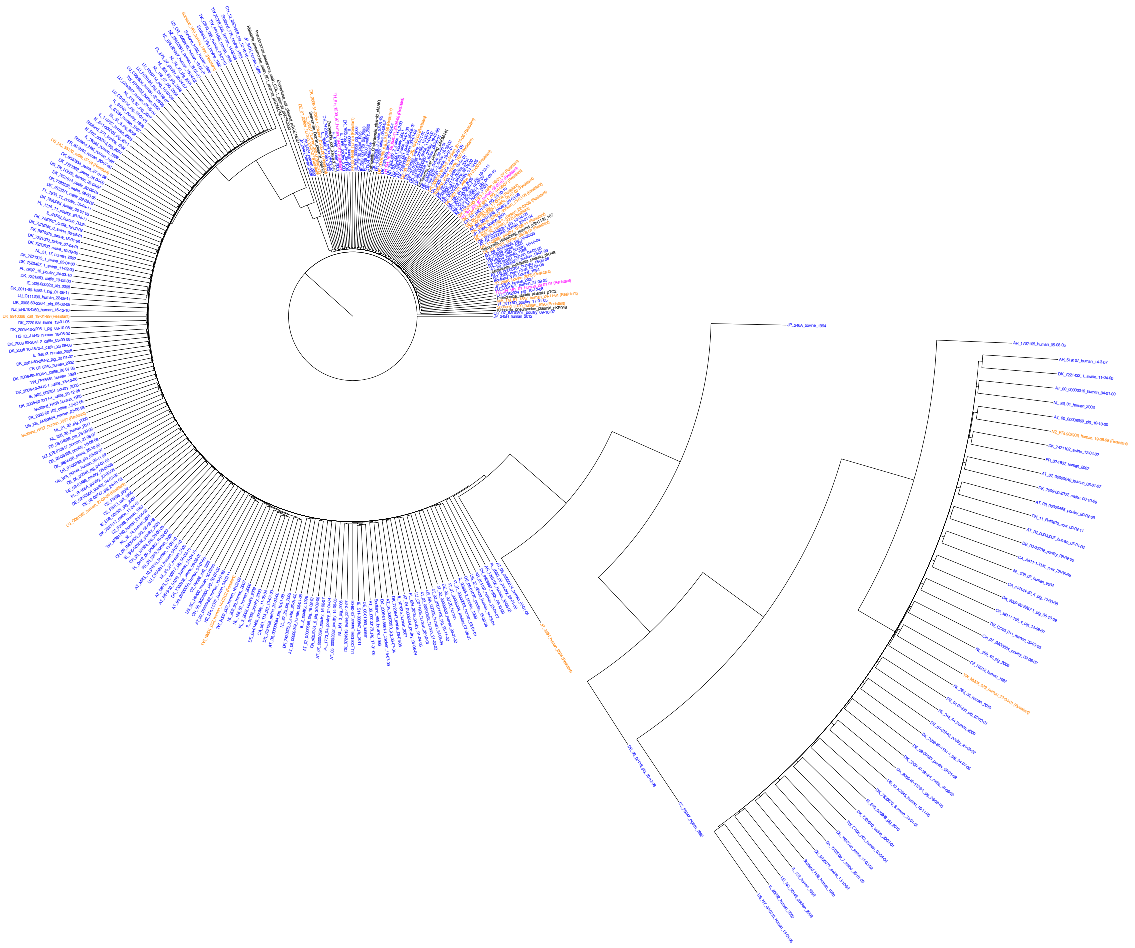

Fig. S11 (D) Maximum likelihood tree of *sul1* genes from DT104 isolates and other bacterial species. DT104 isolates with pink label are resistant strains from susceptible cluster, with orange label are resistant strains from MDR cluster, with blue label are MDR strains. Black labels are other bacterial genomes.

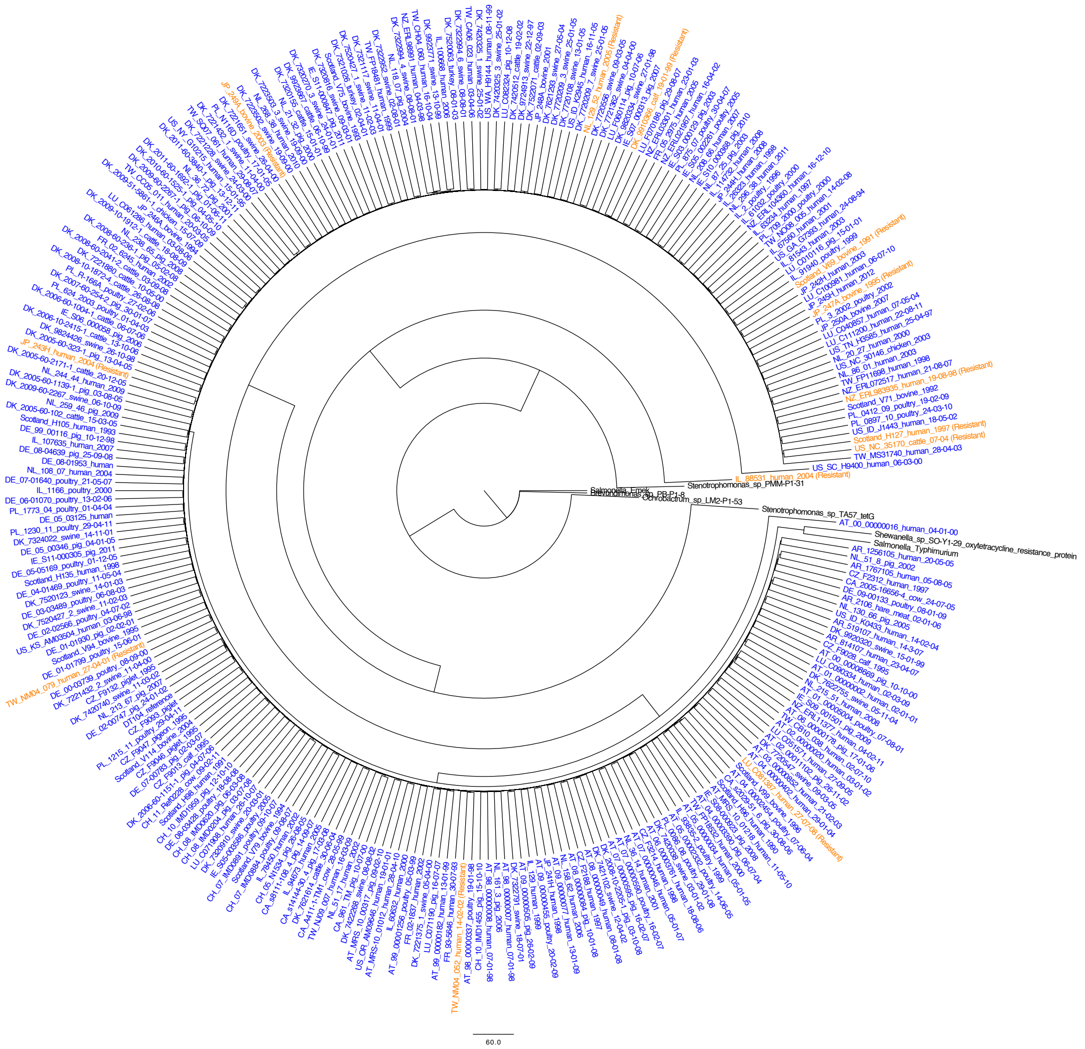

Fig. S11 (E) Maximum likelihood tree of *tet(G)* genes from DT104 isolates and other bacterial species. DT104 isolates with pink label are resistant strains from susceptible cluster, with orange label are resistant strains from MDR cluster, with blue label are MDR strains. Black labels are other bacterial genomes.
